# Philadelphia County Medical Society

**Published:** 1852-05

**Authors:** 


### PHILADELPHIA COUNTY MEDICAL SOCIETY.

*Extra Meeting, April 13th* 1852.

### DISCUSSION ON ANÆSTHESIA.

Continued.

(Reported for the Medical Examiner.)

Dr. Parrish requested that Dr. Patterson would give to the Society the result of his observation on the use of anæsthetics in Europe, he having lately returned from a visit to that country.

Dr. H. S. Patterson said that he should have much preferred to hear a continuation of the discussion of the previous meeting by the gentlemen who so ably participated in it. He was unfortunately not present at that meeting, but he had read the report of its proceedings with much interest and instruction. But, being thus publicly challenged by Dr. Parrish to speak on the subject, he felt it his duty to contribute his mite to the discussion. He had until recently felt, as the great body of the profession in Philadelphia feel, in regard to the anæsthetics. His position had been that of doubt, of distrust, and avoidance of them as a dangerous innovation. These agents came to us in a very questionable shape originally. When the first report of anæsthesia in surgery reached us from Boston, it came, not only startling us by its novelty and the magnitude of the change in practice it contemplated, but also shocking us by its violation of our ethical notions and the savor of empiricism that hung about it. The new agent had a new, high-sounding and unscientific name, and there were rumors of a patent-right to be secured to its discoverers. But when it was shown to be plain sulphuric ether, which any chemist could manufacture and any practitioner administer—when the name of Letheon disappeared, and the letters-patent were no longer heard of—especially when indisputable evidence of its power and utility reached us, we should have given the subject a more favorable reception and examination than we have done. But the mass of resistance to the use of anæsthetics has been more obstinate and impregnable here than any where else in Christendom; and our hospital—alone among great hospitals—has never permitted their employment even in the most painful operations. Participating in these feelings, and never having administered either chloroform or ether in his practice, Dr. P. visited Europe during the past summer, and one of the objects he proposed to himself was to obtain, if possible, more light upon this much mooted question.

In Edinburgh he had the opportunity of seeing and hearing a great deal of the use of chloroform, and it was in regard to the experience of that city that he desired mainly to speak. The undeniable and most important fact is, that in Edinburgh, where chloroform has been used with a degree of freedom, and with a frequency and extent elsewhere unknown, not a single unpleasant circumstance has attended its administration. Every obstetrician, surgeon and dentist in that capital uses it, without exception. Every teacher of obstetrics advises its employment, and gives it to his patients in labor, some in all cases and others only in cases of unusual duration and severity. No surgeon thinks of performing a serious or painful operation, one likely to be attended with great suffering or severe shock, without bringing his patient under its influence. Thousands of patients have inhaled it to full anæsthesia; the chemists manufacture and sell it daily in large quantities; and no one has been injured or even made ill by it. There does not remain a practitioner in Edinburgh opposed to its use. An infinite amount of pain has been relieved, great suffering has been prevented, the dread of operation has been in a great measure removed, and no harm has been done to a single individual. There is no denying these facts. There can be no supposition of concealment or unfair dealing. The character of the gentlemen in whose hands its employment is, of itself precludes the idea as one not to be entertained for a moment. In a large proportion of the cases, if not in all, concealment would be impossible were it attempted. Dr. Simpson introduced the chloroform to our notice as a therapeutic agent, and he bears the unqualified testimony that he has never seen it do any mischief whatever to his patients. Dr. S. is a man whose word, in a matter of medical experience, must be taken without qualification. He has not only the candor of an honest man, but also the discriminating judgment which is not readily deceived, and the calm deliberateness which is not easily led away by the enthusiasm of a hobby. By his kindness Dr. P. was enabled to witness the administration of chloroform to several patients, and to converse with others who had used it in child-birth, some for a considerable length of time. In some cases Dr. P. has maintained its effects for several hours. This powerful agent was always administered with due caution, but with a fearless hand. The patient was thrown into a condition of perfect insensibility, and underwent operations otherwise painful, without any apparent sensation, and certainly with no subsequent recollection of suffering. The manner of Dr. S. indicates the most entire confidence in both the safety and utility of his practice. The lying-in women whom Dr. S. saw were all in excellent health and spirits, and Dr. P. thought that their average "getting up" was shorter and better after chloroform than without it. All asserted that they awoke from the slumber without the slighest after-sensation or recollection of pain, without head-ache or vertigo or nausea, and precisely as after a refreshing sleep. One spirited and intelligent lady, with whom Dr. P. had the honor to converse, roundly asserted that the ladies of Edinburgh *would* have chloroform whether their doctors gave it to them or not; that she would not approach her confinement without it, and that the knowledge that this invaluable boon was within her reach, had taken away all the trembling apprehension which had previously made her pregnancy a period of dread and suffering.

In other parts of Great Britain and on the continent, the anæsthetics are not used so freely as in Edinburgh, but still they are resorted to in all severe surgical operations, and very frequently in obstetric practice. There are very few who use them in every case of midwifery to which they are called, but there are still fewer who do not use them in some cases. No surgeon of any note refuses their employment altogether. They are used in every hospital of London and Paris, more or less. Dr. P. was amused by the ardor of a gentlemen connected with St. Bartholomew's, who, in a conversation on the subject, enthusiastically declared that all the other benefits conferred on mankind by the discovery of America, (bark and our democratic liberty included,) were eclipsed by the inestimable addition of anæsthetics to the treasury of the healing art.

The substance used exclusively in Edinburgh, and most generally elsewhere, is chloroform. The accounts we have of unpleasant or fatal effects from it, do not deter them from administering it. Dr. P. saw no sulphuric ether given, and met with no physician who employed it. The objections to its use are its highly stimulating qualities, (increasing the risk of fever and hemorrhage,) its unpleasant and exceedingly persistent odor, and its liability to leave a disturbed condition of stomach with gastric uneasiness and acid eructations. The question of danger from chloroform he would not discuss at present, except to express his conviction that it is ascribable, in all cases, to some error in the method of administration or in the management of the patient or of the inhaling apparatus. Dr. Simpson gives it on a napkin or handkerchief, lightly folded in a funnel shape and held near the mouth. In obstetrical cases he gives it on the approach of each pain, removing the napkin the moment the relaxation of the patient's muscles shows that the pain is passing off, and not reapplying it until her uneasy motions indicate the approach of another pain and that it is beginning to be felt.

In view of all these circumstances, Dr. P. enquired whether there is not reason to believe that we in Philadelphia have been a little unreasonable in our opposition to these agents. Certain it is, that they alleviate much suffering for which we have hitherto had no remedy, and that they have in a measure taken away the curse of child-birth, and deprived surgical operations of much of their horror.

Dr. Condie was inclined to believe, that a very considerable change, in the opinion of European practitioners as to the employment of anæsthetics, had recently taken place. That a much greater degree of caution in their administration was now inculcated, and that the circumstances under which a resort to them solely to prevent pain, is proper, are admitted to be much more restricted than was the case upon their first introduction. This he inferred, from the very different tone assumed, of late, by the medical journals of Europe when treating of anæsthetics and of the cases in which their administration is justifiable. Much of the wild enthusiasm by which the propriety of a resort to them, in almost every case attended with pain or suffering, was insisted upon by their early advocates, has disappeared. It is now admitted, on all hands, that the leading anæsthetic (chloroform) is an active poison, and that to prevent disastrous consequences following its administration, the utmost caution must be observed; that we are not always able to determine the effects that may be produced, in different individuals, by the inhalation of the same quantity of the article; that the induction of anæsthesia is not justifiable in trifling operations, or in ordinary cases of perfectly natural labor; that, even in those cases in which anæsthesia is considered proper during parturition, it is unnecessary to push it to the extent of inducing an entire abolition of consciousness; and finally, that there are a number of circumstances the presence of which forbids absolutely, in all cases, a resort to it.

Anæsthetics have been in use for too short a period to enable us to arrive at any very certain conclusions in regard to their real value as therapeutic agents—to determine accurately all the cases and circumstances to which they are adapted, or to lay down the proper rules for their safe administration. It unfortunately happens that, whenever any important remedy is announced, instead of a dispassionate examination of its properties, and a cautious testing of its curative powers, it is at once seized upon by a set of enthusiasts, who laud it far beyond its deserts, and ascribe to it remedial virtues under circumstances where it is afterwards ascertained to be powerless for good, if its effects be not even positively injurious; and if the agent be one of great activity, it is only after the infliction of much injury from its injudicious employment, that it attains its proper place upon the lists of the materia medica. When iodine was introduced as a remedy—to believe those who first described its properties and therapeutic powers—it was to remove, at once, consumption from the list of incurable diseases, and to arrest all scrofulous affections, with as much certainty as the bark or quinine is known to arrest the paroxysms of intermittent fever.

But, in reference to no remedial agent, has this wild enthusiasm been displayed to as great an extent perhaps, as it has in regard to anæsthetics. By their agency pain was to be expunged from the catalogue of human evils—child-bearing was to be divested of its agony, and the knife of the surgeon was to remove the diseased member from the body without the patient suffering a single pang, and this without the slightest danger or inconvenience either immediate or remote.

No wonder need be experienced that anæsthesia should have secured from the very first so many ardent advocates, that it should have been recklessly resorted to in almost every operation, in every case of labor, and in almost every disease attended with pain or acute suffering of any kind. Nor has, as yet, the occurrence of death after death, directly attributable to the influence of the agents employed in its production, been a sufficient warning to deter all from a resort to these, merely for the prevention or relief of pain.

By the physician, pain is to be studied with very different feeling from those prompted by a morbid sensibility. Its removal may not, under all circumstances, be advisable. Though invariably an evil, still, cases may certainly occur, in which it would be better to allow the patient to endure, for a short period, even severe suffering, than to induce in him a state of complete insensibility. It was the opinion of Dr. Physick, that, generally speaking, important operations are more successful in those patients who give full vent to their feelings, than in those, who, by a powerful effort of the will, suppress all indications of suffering. And there is some foundation for the enquiry whether, even admitting that, by the employment of anæsthetics, surgical operations may be divested of pain without any immediate danger to the life of the patient, the ultimate result of such operations is as favorable, upon the whole, as of those in which anæsthesia has not been induced.

A comparison that has lately been made by an acute and accurate observer, in our midst—based on the statistics of several large hospitals—of the results of operations in the same institution, before and after the introduction of anæsthetics, as well as the results of operations performed in those hospitals where anæsthetics are now invariably employed, with that of the same class of operations performed in the Pennsylvania Hospital, without the induction of anæsthesia, would lead us to suspect that the abolition of pain by anæsthetic agents does actually exert an unfavorable influence upon the result of operations; an opinion at which Dr. Porter of the U. S. army had arrived from his experience of the use of these agents, during the war with Mexico.

In obstetrics, Dr. Ramsbotham, in the last edition of his *Obstetric Medicine and Surgery*, has denounced the employment of anæsthetics, excepting in some few exceptional cases; while Dr. Chowne, in a late discussion before the London Medical Society, remarked, that he had seen most disastrous consequences follow the administration of chloroform—not only simple after-consequences, but serious mental disorders. The fact of the occurrence of insanity from the use of chloroform is insisted upon by Dr. Webster, who has adduced several cases in which such a result occurred; his statements are of a character that forbid our treating them with entire indifference.

But even if we could, with positive and invariable safety, prevent the pains of parturition by the employment of anæsthetics —would it always be proper to do so? There may be some practitioners of such consummate skill in the application of obstetrical instruments, who, directed solely by their knowledge of the anatomy of the pelvis, and the position of the head, can apply them, without any risk of injury to the organs of the female, as well while she is in a state of entire unconsciousness as when she is in a condition to advise him, promptly, of any pain he may inflict upon her. Dr. Condie, however, very freely confessed that he preferred always, in the application of instruments during labor, that the susceptibility of the female to painful impressions should be unimpaired, as an additional safeguard to prevent her suffering injury from maladroitness on his part.

Much surprise has been expressed by Dr. Parrish, that the comparatively few cases in which fatal effects have resulted from the employment of anæsthetics should be used as an argument against a resort to them in surgical operations and during labor, when the same objection will equally lie against the administration of any of the more potent and useful articles of the materia medica. But is it not far more surprising that the gentleman should not see the very material difference between the employment of a dangerous remedy to *save life*, and a resort to a similar remedy *merely to destroy pain*, in a case that we have every reason to believe, so far as the safety of the patient is concerned, will do very well without it? It will not do to point to the 9000 operations safely performed at St. Bartholomew's Hospital under the influence of anæsthetics, in evidence of the propriety of resorting to it, as a mere preventive of pain—for the one single case of death acknowledged to have occurred in that institution from the use of anæsthesia, independently of others of which we have been informed, is sufficient to counterbalance all the good supposed to have resulted from it, by the mere abolition of pain, in the 9000 cases. The life of one patient in 10,000 ought not to be sacrificed to obtain for the remaining 9999, relief from what is, in fact, in most cases, a very short period of suffering.

Dr. C. remarked, that he did not wish to be considered as altogether opposed to the employment of anæsthetics as therapeutic agents. He was satisfied that their introduction had armed us with a most potent and valuable means of relief in a numerous class of cases. From their internal use, by inhalation, as well as from their application externally, he has derived the most beneficial results in many instances: he has seen them arrest, immediately, the most violent paroxysms of puerperal convulsions, allay promptly the intense suffering induced by cramp of the stomach and intestines, and that attendant upon neuralgic affections of the internal organs, and of various portions of the surface. He could even conceive of operations in which the risk of danger from their employment, should not deter us from availing ourselves of their pain-abolishing effects. An operation may be necessary to save life, but one, at the same time, attended with so much and so long continued suffering, as to cause the patient to hesitate between its endurance and the *certainty of death* should it be omitted or delayed. In such a case, Dr. C. would not hesitate to recommend the induction of anæsthesia—believing that the risk the patient will incur from it will be fully compensated by its enabling him to undergo an operation, the pain of which it would, otherwise, be scarcely worth enduring to purchase the few additional years of existence it may possibly bestow. Cases too occur, not necessarily fatal, of violent paroxysms of pain, occurring periodically, at shorter or longer intervals, and during a very long period of time, where relief from suffering, by which life is rendered a burden, is cheaply purchased by any remedy, even one, the exhibition of which may be attended with considerable danger. Such cases are, unquestionably, proper ones for the employment of anæsthetics; under many other circumstances also, they will no doubt be found useful and judicious remedies—even although no other good effect should attend their exhibition than the relief of pain.

Dr. Parrish remarked that he was surprised to find the statistics of Hospitals introduced here as opposed to anæsthesia in large surgical operations. The basis of the calculation, as I understand it, is a comparison between the results of amputations in the hospitals of New York and Boston, since the introduction of anæsthetic agents into these institutions, and those of the Pennsylvania Hospital, where they are not used. Dr. Condie, who generally speaks with such accuracy, on questions when facts are concerned, has not brought forward the figures by which he claims to sustain his position, but has made a general statement that a comparison between these institutions is altogether unfavorable to the use of anæsthetics.

Now, will any one undertake to say that a slight difference in the percentage of mortality, after operations of any given class, running through a period of only a few years, and without taking into account all the attending circumstances, the comparison being made between three institutions, and those not of the largest class, is to be taken as evidence against the use of an article, which has been employed thousands of times in the largest hospital establishments in the world?

Why, it is well known that the statistics of a hospital bearing upon any given point, may vary very much from year to year, and that a series of unfortunate results in one or two years will influence very materially the general results for a series of years. Thus an epidemic erysipelas may prevail, and carry off a large number of patients who have been subjected to capital operations; and if the institution should be visited with such an epidemic for several seasons in succession, the mortality may become so heavy, as to keep it in the rear of the more favored institutions for a long series of years. There are, too, various other circumstances which may influence the mortality, entirely independent of the use of anæsthetics, and which in my judgment would render it very unsafe to draw conclusions, based upon the experience of two or three institutions during a period of a few years. I apprehend, also, that a difference will be found in the percentage of mortality after any given capital operation, during a series of years, amongst the best hospitals of this and of other countries, both before and since the introduction of anæsthetic agents, showing that other causes are constantly varying the results.

But if a difference in the mortality of hospitals prior to, and since the introduction of anæsthetic agents, is to be taken as an argument for or against them, we have a much more extended field of comparison than that offered by the three chief hospitals of this country.

The large hospitals of Europe furnish much more reliable data; and it so happens that the advocates of anæsthesia point triumphantly to the statistics of these institutions as affording ample evidence of a diminished mortality after the great capital operations, since the introduction of these pain destroying agents.

In the elaborate work of Professor Simpson, on anæsthesia in Surgery and Midwifery, the reader will find two chapters devoted to the question, whether anæsthesia increases or decreases the mortality attendant on surgical operations. The author has here collected the statistics of mortality after the large amputations, in different countries, as derived from official sources, including Phillips' tables, which comprise not only the results in hospital practice, but in the private practice of physicians in Great Britain and in other countries, so far as recorded in the periodical literature of these countries. He has also availed himself of Malgaigne's tables of results from the Parisian hospitals, forming altogether one of the most complete and extensive collections of cases yet published. The percentage of mortality after large amputations, calculated from these data, which were formed prior to the introduction of anæsthetic agents, are then compared with the results after the same operations with anæsthetics, as derived from the

British and Parisian hospitals, forming a series of upwards of 300 cases, up to the time when Professor Simpson wrote.

The conclusion arrived at is, that the mortality since the introduction of anæsthesia is sensibly diminished, and the argument in favor of anæsthetics thus derived is considered by Dr. Simpson as most cogent and convincing. I can only refer the members to this array of figures on the Ether side of the question, and ask them to compare them with those now brought forward on the other side, and see which is the stronger.

I believe myself that there is some degree of uncertainty in this method of calculation, but if it is relied on, Etherization has evidently the advantage.

In regard to the recent unfortunate death from chloroform at St. Bartholomew's hospital—it is certainly against it—though it is only one death in over nine thousand cases, in which the article has been administered there; still, this with the others which have been reported, shows that chloroform is a most potent agent, and that it should not be given on trivial occasions, and without great caution, while at the same time one death in ten thousand should not cause us to abandon it. I myself have always preferred the ether, believing it to be safe and reliable, and should not use the more powerful agent, where this answered the purpose.

Dr. Hays, stated that he was not disposed to discuss, on the present occcasion, the question of the propriety of using anæsthetic agents; but the statements made this evening by the gentleman who opened the debate, relative to the general and indiscriminate use of chloroform in Edinburgh, without a single instance of injurious effects resulting therefrom, were calculated, he conceived, to convey the very erroneous impression that no risk attended the employment of that potent agent. He felt impelled, therefore, by a sense of duty, to put in a word of caution. Nearly thirty cases in which death has been produced by the administration of chloroform have been recorded with their full details; and perhaps double that number have been casually referred to in debates before different learned bodies, but the particulars of which have never been published. Not a few cases also of death after the administration of chloroform have occurred, in which, he believed, the result was chargeable to that agent, though ascribed to other causes. Be this, however, as it may, he thought that undoubted cases enough might be brought forward to demonstrate that the employment of chloroform was not devoid of danger.

Some of the recorded cases of death from chloroform have been ascribed to the article employed being impure; others to carelessness in its administration; and others again to some constitutional peculiarity or organic disease which should have forbid its employment; and thus it has been attempted to prove that in all these cases the fatal result might have been obviated by proper caution.

A case of death from chloroform, however, has very recently occurred which does not allow of any such loophole to escape, from the fact that danger always attends the use of chloroform. This case occurred—where of all places it should have occurred in order to teach a salutary lesson of caution—in St. Bartholomew's Hospital, London—where it had been boasted that chloroform had been used in between nine and ten thousand cases, in not one of which "has its employment left a stain on its character as an innocuous agent of good."

The subject of this case, was a young man only 23 years of age, affected with aneurism by anastomosis implicating the ear and a portion of the scalp behind it, and which had commenced when the patient was four years of age. He was admitted into St. Bartholomew's Hospital, and Mr. Lloyd determined to attempt its cure by ligating the vessels supplying it. Chloroform was given to the patient, and he was kept under its influence for half an hour, the operation having proved a tedious one. He recovered from its influence without any ill effects. The tumor was not, however, entirely obliterated. A little over a month subsequent to this operation, Mr. Lloyd found on examination an artery between the angle of the jaw and mastoid process which pulsated strongly, and on compressing it so as to arrest the circulation in it, the pulsation in the tumor entirely ceased. Mr. Lloyd determined to apply a ligature to it. Chloroform was again administered by the careful and experienced assistants of the Hospital, and the article used was from the same bottle which had been administered in the previous operation. In from five to ten minutes he came under the influence of the article, and Mr. Lloyd commenced the operation. Scarcely had he incised the skin when his assistant informed him, that the patient's pulse was sinking, and before any restoratives could be applied, he ceased to breathe. Every means that could be devised were resorted to in order to re-animate him, but without success. On post mortem examination, no morbid organic change in any organ was found to explain the fatal occurrence; but the blood appeared to have been poisoned. "It was fluid, and it remained without coagulation after its escape from the heart and vessels. It had also a brownish purple hue, much like that which is commonly observed in the spleen; none of it, when thinly spread out, presented the ordinary dark, black or crimson hue of venous blood." But for this untoward administration of chloroform, the patient might have lived to the usual term of human existence. Here, then, is a case in which no human foresight could have provided against the fatal result. The chloroform used was pure, and had been employed before without any ill effects; there was no idiosyncrasy in the patient forbiding its employment, for he had previously been under its influence with apparent impunity; that there was no want of caution and skill in its administration, we have the assurance in the fact that it was given by the experienced assistants of the Hospital; and that there was no organic disease forbiding its employment, was demonstrated by the autopsy. If this case is not calculated to prove that danger attended the administration of chloroform, Dr. Hays said he would be at a loss to know what kind of evidence gentlemen required for that purpose.

In reply to an inquiry made by Dr. Parrish, whether he (Dr. Hays) considered the case he had alluded to forbiding the use of chloroform in every case, Dr. H. stated, that were he compelled to undergo a very painful and protracted operation, he would himself take an anæsthetic, preferring to run the risk rather than endure long and severe suffering, and he would allow his patient the same option, if, after fully explaining to him the state of the case, he should desire to do likewise. But to resort to chloroform for every trifling operation, and in all cases where the slightest and but temporary pain was to be endured, he conceived not only to be injudicious, but actually criminal.

P. S. Since these remarks were made two more cases of death from chloroform have occurred, one in Boston, the other in New Haven.

Dr. H. S. Patterson remarked that the meeting had got pretty well into the discussion of the question on its merits, when Dr. Hays brought down the St. Bartholomew's case upon us as a sudden extinguisher, and it appears that we cannot get beyond it. Around that all the discussion seemed now to centre, and it must be got rid of before any further progress could be made. He did not know that he could get rid of it, but at all events, it could be looked fairly in the face, and its real importance determined. The idea that arose in his mind while Dr. H. was speaking, was that this, like other cases of alleged death from chloroform, came with an air of mystery about it. A mischief has been done, somehow or another; life had been extinguished in some way; but the only fact now apparent is that the chloroform has done it. Let this be admitted, and it does not necessarily follow that the agent in question cannot be used at all therapeutically. The conclusion is greater than the premises will bear. The superstructure is much too large for the foundation. The fact seems to be that chloroform may be—indeed has been—inhaled in such manner as to produce death. Every article of the materia medica of any value, is toxical or at least injurious in some method of exhibition or some dose. Generally, the toxical is in the direct ratio of the therapeutic power. The potency which is curative in its judicious employment, may be deadly otherwise applied. The mere fact that a medicinal agent is capable of destroying life is no argument against its use. Is there any substance of acknowledged power in our pharmacopœia that may not do mischief? Are there not many that have been fatal to human life, not only when used criminally, but also injudiciously employed, although with the best intentions? He (Dr. P.) is not one of those who would willingly uncover the nakedness of the profession or expose its shame, but he would ask whether chloroform has destroyed more human lives than opium, even in its medical use? He would only refer to the old treatment of *delirium tremens* by heroic doses of that drug. He had seen patients die in that disease, in the Pennsylvania Hospital and in the Alms-House, and who would say whether the condition which preceded death was coma or fatal narcotism? He had his own convictions on the subject, and they were such as to lead him to seek a mode of treatment for that affection without opium. But, admitting all this, does it prove that opium should not be used? On the contrary, opium remains an indispensable portion of our means of cure in innumerable cases. If a substance is poisonous, it is so in certain doses, in a particular mode of exhibition, and according to fixed and ascertainable laws. We can determine by observation what functions it affects, in what quantities and in what way. We do not hesitate to use poisons much more deadly than chloroform is alleged to be by its most vehement opponents, because we know their action and can regulate it to good therapeutic ends. If a patient should die of a dose of hydrocyanic acid or strychnine medicinally given, there would be no hesitation in concluding that there had been a gross error in the dose or in the manner of exhibition. Why refuse the application of the same rule to chloroform? It may be asserted that its poisonous influence is so subtle and so fatal that it cannot be regulated. But this is disproved by thousands upon thousands of cases of its successful administration. If it is poisonous at all, it is so only in a certain quantity or by a certain rapidity of introduction, which can be clearly ascertained, scientifically regulated, and securely guarded against. Let us now look, with a more thorough scrutiny into the St. Bartholomew's case, which has been thrown in our way as an impediment not to be got over. The first fact to be noticed is that this same patient, not a month previous, was kept under the full influence of chloroform for twelve minutes, during a painful operation, and without the slightest inconvenience or interruption to his recovery. There was plainly no "idiosyncrasy" here. It is proved that chloform could be administered to that very patient with safety and with the most beneficial results. On the last and fatal occasion, he inhaled the vapor of chloroform for a much shorter time, and died before the first incision was completed by the knife of the surgeon. Now, can any man believe that the chloroform was administered in precisely the same way, in the same quantity, and to the same extent, as on the former occasion? Like causes produce like effects. The man who takes a grain of opium to-day with beneficial effects, will not be fatally poisoned by a grain of opium a month hence. The probability is that in the first instance the chloroform was properly administered with a due admixture of atmospheric air, while in the latter it was hastily presented, of full intensity and undiluted. The quantity to be estimated is not altogether that poured upon the napkin or introduced into the inhaling apparatus, but that actually received into the lungs of the patient, and absorbed from their mucous membrane. A better case for the illustration of the principle just laid down could not be desired. The blame rests with the erroneous mode of the use, and not with the substance used. As for the mere allegation of toxical power, Dr. P. would give very little for a medicine that could not produce such effects in any case. He suspected the efficacy of every agent whose powers were so feeble as not to render it noxious in its inappropriate or immoderate employment.

As to the remarks made by Dr. Condie in reference to the enthusiasm of the advocates of new measures, Dr. P. thought that such a charge in reference to anæsthetics was singularly out of place in Philadelphia. The error here seemed to be all on the other side. He did not wish to complain of the conservatism of the profession here generally. Their cautious skepticism in regard to medical novelties had its great and lasting benefits. But these anæsthetics are no longer a novelty. Their precise value has not been definitely determined, nor have we settled all the laws that should regulate their use; but that they have great and important uses can no longer be doubted. It appears certain that they will become a fixed portion of the armament of the surgeon and the obstetrician. Why should we in Philadelphia alone occupy this position of dogged resistance and refuse to receive them? Dr. Condie has warned us against the enthusiasm of novelty. Dr. P. acknowledged the truth and value of his remarks. There are *quidnuncs* in the profession as well as out of it, and they will run wild after new hobbies. But he would remind Dr. C. that this is not the only dangerous enthusiasm. There is another that, in its relation to other matters, has been recognized and pretty well comprehended in our country, where it has received the generic title of *old-hunkerism.* It consists in an obstinate conservatism, which brands every new thing as an innovation, in the bad sense of the term, without waiting to see whether it may not turn out an improvement. It rests content with old things, it wants no progress, and it resists all new things as essentially evil or mistaken. He was afraid that there was a leaven of the enthusiasm of *hunkerism* in this resolute opposition to the anæsthetics. At all events, the truth must soon make itself manifest, and the fact of the matter be established. There can be no doubt that anæsthesia will become, when better understood, a well regulated and well established portion of our practice, and the most the Philadelphia profession can claim will be the merit of having been the drag on the wheel that prevented a too rapid attainment of the goal.

Dr. Bell, in reply to a question from the President, (Dr. Jackson,) said that Dr. Mussey, his former colleague in the *Ohio Medical College, and the Commercial Hospital of Cincinnati,* uniformly made use of a mixture of ether and chloroform for the subjects on whom he operated ; and without, it is believed, in any case, sinister results.

Dr. Atlee said: There is one point in the case mentioned by Dr. Hays that, perhaps, may have been overlooked. If we take up any book on Surgery, and refer to the subject of operations on the neck, we will find that instantaneous death occasionally results during the operation, and that this is attributed to the entrance of air into the veins of the neck. The question, then, would naturally arise, Could such an occurrence have taken place in this unfortunate case, and death have resulted from this cause and not from chloroform? Always in dread of such an event, I have never administered anæsthetics in important operations on the neck, lest death accruing under such circumstances, might be erroneously attributed to chloroform, and thus impair the character of a very valuable agent.

Dr. Page remarked that, notwithstanding the current of the argument, the opinion of all present on the subject of the use of anæsthetics seems, "like a handle of a jug, all on one side." All of the gentlemen, including those who spoke on the negative side of the question, admit that under certain circumstances these agents may and even ought to be administered, while under other circumstance they should be repudiated as likely to be productive of injury. What then are the circumstances requiring their employment? and the answer is, who can tell? The opponents of their indiscriminate use employ them under very opposite conditions. We are told by some that they should not be applied in cerebral excitements, in disease of the heart, and in pulmonary affections, &c.; but Dr. Condie has used the anæsthetic in convulsions with marked benefit; and in asthma and difficulty of breathing, ether has been highly extolled; and it is very far from probable that, some of the many thousands who have been placed in a state of anæsthesia for surgical operations, the extraction of teeth, &c., and who have done well, should not have been at the time laboring under severe cardiac affection. Now, for my part, I consider that the chief use of the agent, and the principal cause requiring its administration, is for the relief of pain; but to its indiscriminate use I am as much opposed as any member of the profession. I regret that so few of the members here speak from the book or card. They give us the experience and observations of others and not of themselves, and we know that there are few subjects on which there has been so much written and to so little purpose, as on anæsthetics, within the last few years, except perhaps cholera. With me, after a principle is once adopted, it matters not where it comes from, nor does it require a great accumulation of evidence for its elucidation; and I look upon it as a fixed fact that the anæsthetic may be administered whenever severe and prolonged pain would be otherwise suffered, unless strong indications exist to the contrary. None of us would recommend a patient to be placed under the influence of sulphuric or chloric ether or chloroform for a trivial surgical operation, or for the extraction of a tooth, nor would we use them on ourselves, because the pain is soon over, and it is not worth while to run any risk; and in this sentiment I heartily concur, as risk there is; as we know that unfavorable results have followed their administration; and strange as it may seem, many of the fatal cases have occurred after the performance of what are commonly called slight operations. If I recollect aright two fatal cases occurred in quick succession in New York; one when the operation was for fistula in ano, and the other for hemorrhoids, both very simple and every day operations in surgery; and we were thrown into the utmost consternation that human life should have been thus endangered, and could scarcely believe that the result was due to any other cause than the chloroform. But, sir, some of the most simple operations occasionally terminate fatally, without any discernible cause. I once saw a patient lose her life from the passage of a thread through a small vascular tumor in the bend of the arm; and I also saw M. Velpeau remove a small tumor from the shoulder of a woman, and she died in a few hours without any assignable cause. Now, sir, patients will die after operations, and sometimes suddenly, and the surgeon cannot with all his ingenuity account for the death, unless it be ascribed to the luckless anæsthetic.

But it is for the relief of pain during the performance of the more important operations only, as has been before remarked, that it should be administered, and here it not only relieves great suffering, but may have done away with past anxiety, and may remove or diminish future danger. The dread of pain is one of the most depressing and distressing influences to which the suffering patient, or one who is about to submit to a severe operation, is liable. Now if this can be removed, much is gained. The patient who has once made up his mind to the necessity of an operation, does not fear its ultimate results or consequences. If it is one which, of itself, compromises life, the mind becomes settled as to its necessity, and the great fear is of the pain which must be endured during its performance, and the great objection made is that it will *hurt*. Now all of this feeling yields to the confidence which the anæsthetic inspires, and the common question which the patient puts and has always put to the surgeon is, "can you not give me something to relieve the pain?" We all know how intimate the relations are between the mind and body; and if we keep in view the old adage *sana mens in sano corpore*, the whole matter will be fully understood. The mind and nervous system being placed at ease under the belief that no pain will be experienced during an operation, no matter how severe, will diminish much the dangers of what all surgeons have too well known as the nervous shock, or that condition in which patients die from the immediate effect of operations. It was formerly a common thing to hear of persons dying on the table during an operation; but who has known this to occur in Philadelphia when ether has been administered, and we are all aware that it has been given extensively and almost indiscriminately?

Another argument against its use is that the plasticity of the blood is altered, and that the healing process will not take place so readily as when the agent has not been employed. Facts seem to prove the contrary. I have never seen the cure more tardy after the use of ether than without it, and indeed in some instances which have fallen under my observation, recovery has taken place with remarkable rapidity. I assisted Dr. Goddard in an amputation of the thigh after a gunshot wound of the knee-joint, when the injury was tremendous, and when all the circumstances were so unfavorable as to induce me to oppose the administration of ether, not because I thought that it would do harm, but because I thought that the patient would most likely die, and that the fatality of the case would be ascribed to the agent. It was however given under the advice of Drs. Jewell, McClellan, and Wilson of Bustleton, and the only unpleasant consequence was a slight vomiting and faintness. The wound healed, and the old gentlemen is now well, and an approver of ether from experience. I have performed capital operations when the patients have been under its influence, and I have not been satisfied that it has ever proved injurious. I amputated a man's arm, and on the fourteenth day he was well enough to elope from the almshouse. I have removed thighs, legs, the penis and the testicle, with the patients in an unconscious state, and they have all done well. I operated for strangulated inguinal hernia on a patient of Dr. Naudain, to whom chloroform had been previously given freely during the trial of the taxis, both by Dr. N. and myself; and at my visit on the thirteenth day, I received a message that the man had waited all the day before for me, and that he had gone out to walk. These cases and others in my own practice, besides a vast many which have fallen under my observation in the hands of others, make me believe that the agent may be often used without injury, if not with advantage. I lost a patient eighteen months since, after the operation for hernia, when chloroform was employed, but I could not ascribe the death to the agent. I have lost others both before and since, when no anæsthetic was given.

It has been said that the color of the blood is changed, and that black blood flows from a wound after the use of anæsthetics, This I have not observed. After amputations when the arteries have been secured, which is immediately done, the tourniquet is partially loosened, and then it is that the black blood flows, but it comes from the veins and not from the arteries, and the observer, who is perhaps anxious to view it in this light, mistakes the one for the other; but when the constriction above is entirely removed, all bleeding usually ceases.

In medicine anæsthetics have been extensively employed, and even by those who repudiate them in surgery, without disadvantage, and many have been rash enough to use them freely in obstetrics.

In midwifery I have used ether with the view of promoting relaxation of the os uteri, vagina, and the neighboring parts, and to induce a free secretion from *the parts*, which is the harbinger of the coming good time to the patient and accoucheur, and so far I have had abundant cause to be satisfied with its use. It promotes secretion and induces relaxation, while it does not interfere with uterine contraction or the bearing down effort. This is, I know, contrary to theory, but I believe it to be the fact. It seems, as has been well established, to act especially on and to control the voluntary muscles. Now the patient should not be placed in a state of full insensibility, but should be so far affected as not to suffer pain; and while thus influenced the sympathetic or synergic action of the abdominal muscles and diaphragm will be readily called into play by that of the uterus, which acts involuntarily. We may, indeed, say that a patient suffering no pain will readily yield to this sympathetic action, and will bear down forcibly, when without a freedom from suffering, she would be almost unable to follow the assiduous directions of her accoucheur to assist herself. A remarkable case of this kind fell under my care a short time since, in the person of a young lady in her first confinement, who had been suffering active pain from 7 A. M., to 4½ P. M., without making further progress than the dilatation of the os uteri to the size of a quarter dollar. She suffered much and cried loudly for something to relieve her. She inhaled ether; the dilatation took place speedily, *the parts* became relaxed and moist, she bore down *like a woman*, and before 6 o'clock the child was dressed and the mother bandaged up. She expressed herself as comfortable, and so she continued. I have never yet witnessed the occurrence of any unpleasant accident from the administration of ether during labor, and I have seen much suffering avoided.

We are told that it should not be given when instruments are to be applied, and for the reason that the practitioner should be advised by the patient when he is giving her pain and when he is doing injury. This is to a certain extent true, but obstetrics should teach the practitioner how to apply his instruments, and how to avoid mischief without depending entirely on the assistance given by the woman's silence or moans. Who ever yet pulled on the forceps without giving pain, and who has desisted because his patient cried out? Other indications do and should guide him; he should know when and how to operate, and should not trust entirely to his patient for the success of his practice. Nervous irritability may be mitigated in obstetrics as well as in surgery, by the timely and proper use of different remedies, and if we resort to ether instead of opium and its preparations, and other narcotics, it is because it is more efficacious and not more injurious. Every surgeon and every accoucheur will sometimes give the different narcotics. As before remarked, the full anæsthetic effect should not be induced; enough of the agent should be given only to relieve the pain, as it may have to be used for a considerable time.

One strong expression is that ether is intoxicating, but this must depend on the meaning of the term intoxication. If the soothing effect of opium, hyoscyamus, lactucarium, &c., or perchance their exciting or poisonous effects, or the phenomena attending convulsions, coma, concussion, &c., can be called intoxication, then the anæsthetic is intoxicating. We know that the effect of ether is transient and soon passes off, so that after its administration sensibility soon returns; but how soon would a young lady be able to leave a dentist's chair and go into the street after an inebriation from brandy or wine. The effects of drink do not pass off thus suddenly, and etherization is not intoxication.

I do not wish to advocate the use of anæsthetics under all circumstances, nor their indiscriminate application. One circumstance contra-indicating their employment, although other things may call for them, is when it is absolutely necessary to keep a patient perfectly still, as during the operations for hernia, cataract, and others of equal delicacy, when a slight involuntary movement might cause a cut to be made where it should not be made. It should not be used in operations in the mouth, because the patient might not be able to get rid of the blood, and might suffer from its passing downwards. Other contra-indications must constantly arise in particular cases, which should be duly considered by the practitioner. Of this I am convinced, that it is not very easy to kill a person with ether, else death would have occurred in very many instances, for no agent has been so widely and indiscriminately used, and yet with so few bad consequences. Ether should be preferred to chloroform. It does not act so suddenly nor so powerfully, and experience teaches that it is the safer agent. A mixture of the two is used, but I think it objectionable, as ether is the more volatile, and if its effect is not speedily induced it will soon evaporate and leave the chloroform behind to have its full effect. The better plan would be to give ether alone, and then, should the patient be insusceptible to its action or become excited, a few drops of chloroform may be administered. Under all circumstances, a full supply of air should be allowed, and the more simple the apparatus employed, the better.

